# Editorial: Cell death in innate immunity and inflammatory diseases

**DOI:** 10.3389/fimmu.2025.1635279

**Published:** 2025-06-10

**Authors:** Malvina Pizzuto, Chiara Cipollina

**Affiliations:** ^1^ Institute for Molecular Bioscience, The University of Queensland, Brisbane, QLD, Australia; ^2^ Ri.MED Foundation, Palermo, Italy; ^3^ Institute for Translational Pharmacology (IFT), National Research Council (CNR), Palermo, Italy

**Keywords:** regulated cell death (RCD), pyroptosis, necroptosis, ferroptosis, gasdermin

Regulated cell death is essential to maintain tissue homeostasis during normal development as well as to mount effective responses against pathogens (1). Apoptosis, pyroptosis, ferroptosis, and necroptosis have been the most investigated types of regulated cell death, although new pathways continue to be discovered (1). Apoptosis is associated with mechanisms that minimize immune response. By contrast, ferroptosis, necroptosis, and pyroptosis are highly immunogenic since they are lytic forms of cell death, accompanied by the rupture of the cell membrane due to lipid peroxidation, or to the formation of pores by gasdermin (GSDM) or mixed lineage kinase domain-like pseudokinase (MLKL), respectively. In the context of infections, lytic cell death acts as a host defense mechanism to block pathogen replication (2). However, cell lysis causes the release of alarmins and damage-associated molecular patterns (DAMPs), which propagate inflammation. Dysregulation of cell death contributes to susceptibility to infections, tissue and organ injury in inflammatory diseases and, in the long run, may promote the establishment of chronic inflammatory conditions like fibrosis (3, 4). A balance between protective and detrimental effects of lytic cell death is particularly relevant in several disorders such as acute kidney injury (AKI) and diabetic nephropathy, as well as in ischemic stroke, where cell death contributes to neuronal injury. Therefore, fine-tuning of cell death appears to be a promising and still unexplored strategy for treating inflammation in several contexts. Understanding the evolutionary conservation and functional diversification of GSDMs provides insight into how these proteins have evolved to regulate inflammatory cell death across species. It may help elucidate the molecular mechanisms of GSDM activation and regulation, opening new avenues for therapeutic strategies that either dampen harmful inflammation or harness cell death pathways to boost immunity, which may be useful for cancer treatment (5).

This Research Topic presents a collection of review articles exploring the significance of GSDMs during evolution as well as the role of regulated cell death in acute and chronic disease conditions, such as AKI, diabetic nephropathy, and ischemic stroke.

Growing evidence has revealed evolutionary similarities in defense-related proteins between multicellular organisms and microorganisms. In “*Evolutionary insights and functional diversity of gasdermin family proteins and homologs in microorganisms*”, Wang et al. summarize recent research progresses highlighting the conserved role of GSDMs in regulating cell death and immunity in bacteria, fungi and viruses. Fungal GSDM (fGSDM) and bacterial GSDM (bGSDMs) participate in defense-related cell suicide strategies, namely, to heterokaryon incompatibility in fungi and abortive infection in bacteria. Similar to their mammalian and fungal homologues, bGSDMs and fGSMDs are activated by proteolysis and, upon activation, bind to negatively charged phospholipids. Of note, each bGSDM protein displays a unique range of pore diameters. GSDM homologs have also been found in poxviruses, where they can interfere with host inflammatory caspases to hijack host defense systems and facilitate viral replication. Further research will be necessary to improve our understanding of microbial GSDMs biological significance and potential.

A consequence of dysregulated cell death may be the worsening of acute diseases or the establishment of chronic conditions. In “*Regulated necrosis role in inflammation and repair in acute kidney injury*”, Guerrero-Mauvecin et al. report recent evidence suggesting a prominent role for ferroptosis and necroptosis in AKI as well as in the progression of AKI to chronic kidney disease (AKI-to-CKD). A key pathogenetic mechanism in AKI is tubular cell death. After injury, tissue repair and regeneration occur. However, when defective, these processes may lead to fibrosis, contributing to CKD progression. During ferroptosis, in addition to DAMPs release, lipid peroxides are massively released and contribute to cell death propagation to adjacent tubular cells, which may result in the whole tubule death. An improved understanding of the triggers and prevalent regulated cell death mechanisms, as well as their connection with inflammatory reactions, may open new therapeutic avenues to treat AKI and AKI-to-CKD transition.

Similar to AKI, ferroptosis has been indicated as a central pathogenic trigger in diabetic nephropathy (DN). In the review “*Ferroptosis: an important player in the inflammatory response in diabetic nephropathy*”, Li et al. highlight how hyperglycemia-driven iron overload and lipid peroxidation may initiate ferroptosis, contributing to renal injury through crosstalk with inflammatory pathways, including cyclic GMP-AMP (cGAS) and the cyclic GMP-AMP receptor stimulator of interferon genes (STING), Janus kinase/signal transducer and activator of transcription (JAK/STAT), nuclear factor kappa B (NF-κB), Mitogen−activated protein kinase (MAPK) and inflammasomes. The review also explores the role of mitochondria as both a source of reactive oxygen species and a hub for ferroptotic signaling. The authors also discuss how ferroptosis can either promote inflammasome activation or suppress it, as certain lipid peroxidation byproducts like 4-Hydroxynonenal may directly inhibit activation of the NLRP3 inflammasome.

The cGAS-STING pathway is emerging as a key regulator of inflammation and cell death in several pathological contexts. In the review “*Novel insight into cGAS-STING pathway in ischemic stroke: from pre- to post-disease*” Ma et al. explore the role of the cGAS-STING pathway in stroke pathophysiology. The authors examine how metabolic dysfunction, mitochondrial damage, and the release of mitochondrial DNA activate cGAS-STING signaling, promoting microglial-driven neuroinflammation. Importantly, they discuss the emerging links to oxidative and ER stress and regulated cell death mechanisms, including ferroptosis and other types of cell death. The review also illustrates how cGAS-STING exerts context-dependent effects—amplifying or dampening inflammation through redox-sensitive mechanisms. These insights underscore the potential of targeting cGAS-STING to modulate inflammation and cell death in ischemic stroke and inspire novel immunotherapeutic strategies.

The complex and interconnected processes of apoptosis, necroptosis, pyroptosis, ferroptosis, and other forms of regulated cell death are crucial for normal development and maintain equilibrium within the body. However, as summarized by the review articles presented herein, dysregulation of these processes can lead to disease, highlighting the need for tight control. Deciphering the signaling mechanisms that govern regulated cell death has opened new avenues for therapeutic intervention. As an example, there is a growing interest in investigating the potential of inducing proinflammatory cell death to strengthen anti-cancer immunity. Conversely, strategies aimed at dampening inflammatory lytic cell death to reduce the release of proinflammatory DAMPs hold promise for treating inflammatory disorders. Considering the pivotal role of cell death in biology, preclinical and clinical investigations will be crucial in defining how we can modulate these pathways and what may be the impact of cell death modulation on patient outcomes.
